# Vitamin D and the Risk of Depression: A Causal Relationship? Findings from a Mendelian Randomization Study

**DOI:** 10.3390/nu11051085

**Published:** 2019-05-16

**Authors:** Lars Libuda, Björn-Hergen Laabs, Christine Ludwig, Judith Bühlmeier, Jochen Antel, Anke Hinney, Roaa Naaresh, Manuel Föcker, Johannes Hebebrand, Inke R. König, Triinu Peters

**Affiliations:** 1 Department of Child and Adolescent Psychiatry, Psychosomatics and Psychotherapy, University Hospital Essen, University of Duisburg-Essen, 45147 Essen, Germany; christine.ludwig@lvr.de (C.L.); judith.buehlmeier@uni-due.de (J.B.); jochen.antel@uni-due.de (J.A.); Anke.hinney@uni-due.de (A.H.); roaanaaresh@gmail.com (R.N.); johannes.hebebrand@uni-due.de (J.H.); triinu.peters@uni-due.de (T.P.); 2 Institut für Medizinische Biometrie und Statistik, Universität zu Lübeck, Universitätsklinikum Schleswig-Holstein, 23562 Lübeck, Germany; laabs@imbs.uni-luebeck.de (B.-H.L.); Inke.Koenig@imbs.uni-luebeck.de (I.R.K.); 3 Department of Child and Adolescent Psychiatry, University of Münster, 48149 Münster, Germany; Manuel.Foecker@ukmuenster.de

**Keywords:** vitamin D, depression, depressive symptoms, Mendelian randomization

## Abstract

While observational studies show an association between 25(OH)vitamin D concentrations and depressive symptoms, intervention studies, which examine the preventive effects of vitamin D supplementation on the development of depression, are lacking. To estimate the role of lowered 25(OH)vitamin D concentrations in the etiology of depressive disorders, we conducted a two-sample Mendelian randomization (MR) study on depression, i.e., “depressive symptoms” (DS, *n* = 161,460) and “broad depression” (BD, *n* = 113,769 cases and 208,811 controls). Six single nucleotide polymorphisms (SNPs), which were genome-wide significantly associated with 25(OH)vitamin D concentrations in 79,366 subjects from the SUNLIGHT genome-wide association study (GWAS), were used as an instrumental variable. None of the six SNPs was associated with DS or BD (all *p* > 0.05). MR analysis revealed no causal effects of 25(OH)vitamin D concentration, either on DS (inverse variance weighted (IVW); b = 0.025, SE = 0.038, *p* = 0.52) or on BD (IVW; b = 0.020, SE = 0.012, *p* = 0.10). Sensitivity analyses confirmed that 25(OH)vitamin D concentrations were not significantly associated with DS or BD. The findings from this MR study indicate no causal relationship between vitamin D concentrations and depressive symptoms, or broad depression. Conflicting findings from observational studies might have resulted from residual confounding or reverse causation.

## 1. Introduction

Depressive disorders are the most common mental disorders worldwide, with more than 300 million people having been affected, in 2015 [1]. Considering that major depressive disorders (MDD) recently became the third leading cause of disability worldwide [1], effective preventive approaches are urgently needed. Interventions aiming at the prevention of mental disorders should ideally focus on youth, since the first onset of mental disorders is frequently seen during childhood/adolescence [2].

Diet as one modifiable lifestyle factor could be a target for such preventive interventions. Considering the increasing evidence for an inverse association between diet quality and mental disorders, it was recently claimed to consider “nutritional medicine as mainstream in psychiatry” [3]. At the same time, vitamin D deficiency was suggested to be linked with increased depressive symptoms [3]. In fact, a number of studies have confirmed a relationship between vitamin D levels and mental health already in childhood and adolescence. The majority of such studies focused on the autism spectrum disorders and ADHD [4]. The few observational studies on depression in adolescence seemed to confirm the suggested inverse association between 25(OH)vitamin D concentrations (recommended biomarker for vitamin D status), and depression or emotional problems [5,6,7]. However, findings from observational studies are not sufficient to draw conclusions on cause–effect relationships.

In childhood and adolescent depression, randomized controlled trials (RCTs)—the gold standard to imply causality—are currently missing [4,8]. In adults, RCTs have examined vitamin D supplementation effects on the course of an already existing depression, but meta-analyses of these studies have revealed conflicting results [9,10,11]. Furthermore, these RCTs have focused on the therapeutic effects, but have not examined a preventive role of vitamin D, prior to the development of depression. The few RCTs on preventive effects had only been conducted in postmenopausal women [12] or women older than 70 years [13], without showing any beneficial effects. Transferability of these results to the general population remains unclear. However, such preventive RCTs would require long-term interventions and follow-up periods of several years, to cover a critical time frame of disorder pathogenesis, and very large sample sizes, to provide sufficient statistical power. In contrast, two-sample Mendelian Randomization (MR) studies based on summary data from large-scale genome-wide association studies (GWAS) are a time-effective approach to examine the causal effect of an exposure (e.g., 25(OH)vitamin D concentration) on an outcome (e.g., depression), by using genetic markers as instrumental variables (IVs) [14]. The concept of MR studies implies a natural “quasi randomization”, since the individual composition of alleles and, thus, of IVs are determined randomly at conception, resulting in a reduced risk of confounding [15]. Bias from reverse causation, another limitation of observational studies, is also precluded in MR studies, as the individual genotype is determined at conception, and cannot be modified by the outcome of interest [15].

Recently, a two-sample MR study was conducted which focused on the causal effects of 25(OH)vitamin D on MDD. Using data from the most recent MDD GWAS with 59,851 cases and 113,154 controls, this MR study did not reveal a causal association between 25(OH)vitamin D concentrations and the risk of MDD [16]. However, the phenotype of MDD does not completely cover the complex dimensional and transitional aspects in the etiology of depression, ranging from single depressive symptoms to a depressive syndrome, and finally a diagnosis of MDD or other subtypes of depressive disorders [17]. To include both the dimensional aspects of depression and different depression subtypes as outcomes, our two-sample MR study examined the effects of 25(OH)vitamin D concentrations on depressive symptoms (DS) and broad depression (BD), using summary-level data of the most recent large-scaled GWAS.

## 2. Materials and Methods

### 2.1. Data Sources for MR Analyses

This two-sample MR study relied on publicly available summary statistics of three different GWAS meta-analyses on 25(OH)vitamin D concentration [18], DS [19], and BD [20,21]. For the definition of the genetic instrument we used the most recent GWAS on serum 25(OH)vitamin D (i.e., the exposure in this MR analysis) from the SUNLIGHT consortium with 79,366 participants of European ancestry, including 31 studies from Europe, Canada, and USA [18]. The following studies were included in the SUNLIGHT GWAS: 1958 British Birth (1956BC), the Cardiovascular Health Study (CHS), the Framingham Heart Study (FHS), Gothenburg Osteoporosis and Obesity Determinants (GOOD), the Health, Aging, and Body Composition study (Health ABC), the Study of Indiana Women (Indiana), the Northern Finland Birth Cohort 1966 (NFBC), the Old Older Amish Study (OOA), the Rotterdam Study, the Twins UK registry, the Alpha-Tocopherol, Beta-Carotene Cancer Prevention Study (ATBC), the Atherosclerosis Risk in Communities Study (ARIC), the AtheroGene registry, B-vitamins for the Prevention of Osteoporotic Fractures (B-PROOF), Epidemiology of Diabetes Interventions and Complications (EDIC), the Case-Control Study for Metabolic Syndrome (GenMets), the Helsinki Birth Cohort Study (HBCS), the Health Professional Follow-Up Study (HPFS, nested coronary heart disease case-control study), the Invecchiare in Chianti Study (InChianti), the Cooperative Health Research in the region Augsburg (KORA), the Leiden Longevity Study (LLS), the Ludwigshafen Risk and Cardiovascular Health Study (LURIC), the Multi-Ethnic Study of Atherosclerosis (MESA), the Nijmegen Biomedische Studie (NBS), the Nurses’ Health Study (NHS, nested breast cancer case-control study, and type2 diabetes case-control study), the Orkney Complex Disease Study (ORCADES), the Prostate, Lung, Colorectal, and Ovarian Cancer Screening Trial (PLCO), the PROspective Study of Pravastatin in the Elderly at Risk (PROSPER), the Study of Health in Pomerania (SHIP), the Scottish Colorectal Cancer Study (SOCCS), and the Cardiovascular Risk in Young Finns Study (YFS). The majority of these 31 studies used radioimmunoassay techniques, HPLC-MS, or LC-MS for the detection of serum 25(OH)vitamin D. Detailed information about ethical approvals of the respective studies, as well as the sample characteristics, are given in the supplementary files of the SUNLIGHT GWAS meta-analysis [18,22]. For our MR study we used independent single nucleotide polymorphisms (SNPs) of all six loci as genetic instruments, which were genome-wide significantly associated with the exposure of interest (i.e., serum 25-hydroxyvitamin D concentrations) [18]. The effect sizes of the six genetic variants on the exposure were derived from the publicly available summary statistics of the GWAS meta-analysis (Table 1). Overall, these six SNPs explained 2.8% of the variance of serum 25(OH)vitamin D concentrations [18].

Effect estimates of these six genetic instruments on the two outcomes DS and BD were obtained from the summary statistics of the two GWAS, including 161,460 individuals with European descent for DS [19] and 113,769 “white British” BD cases, along with 208,811 controls from the UK Biobank for BD [20,21]. The GWAS on DS [19] used data of the following cohorts: (1) UK Biobank (information about ethics: https://www.ukbiobank.ac.uk/wp-content/uploads/2011/05/EGF20082.pdf.) (2) GERA, Resource for Genetic Epidemiology Research on Adult Health and Aging; Subsample of the longitudinal cohort enrolled in the Kaiser Permanente Research Program on Genes, Environment, and Health (RPGEH) [23]. The institutional review boards of both, KPNC and the University of California San Francisco approved the project. (3) The Psychiatric Genetics Consortium (PGC) data on MDD [24] also relied on the GERA cohorts. The UK Biobank included people who were currently aged 40–69, participants of RPGEH were all adult (≥18 years old). Okbay et al. (2016) considered only persons with European ancestry (UK Biobank: “White-British” ancestry) and constructed a continuous phenotype for DS, by combining the responses to two questions about the experienced feelings of disinterest and feelings of depression with case-control data on MDD [19].

The GWAS on BD [20] used data from the UK Biobank study [25], which was conducted under the generic approval from the NHS National Research Ethics Service (approval letter dated 17th June 2011, Ref 11/NW/0382). According to the study protocol, the UK Biobank aimed to include 500,000 people from all over UK, who were currently aged 40–69 and were living within a reasonable travelling distance to an assessment center. While the study protocol of the UK Biobank (https://www.ukbiobank.ac.uk/wp-content/uploads/2011/11/UK-Biobank-Protocol.pdf) did not list any criterion for exclusion, Howard et al. excluded individuals which were not recorded as “white British” as well as the outliers (e.g., based on heterozygosity, shared relatedness of up to the third degree, etc.) [20]. Howard et al. defined BD via self-reported help-seeking behavior for non-specific mental health difficulties, which might reflect the beginning of a depressive episode [20]. They hypothesized that genetic variants identified in cohorts using self-reported measures of depression are known to be highly correlated with those obtained from cohorts using clinically diagnosed depression phenotypes, but at the same time offer the opportunity to analyze large cohorts rather than smaller studies with a clinically defined phenotype [20]. Accordingly, BD represents the broadest of the examined phenotypes in this GWAS, including different depression diagnoses (e.g., single episode depression, recurrent depressive disorder), which resulted in the largest number of, both, subjects for analysis and identified significant SNPs [20].

Using summary data of these three GWAS, two separate two-sample MR studies were conducted for BD and DS, including analyses of pleiotropy, sensitivity, and power.

### 2.2. Testing Mendelian Randomization Assumptions

MR studies require fulfillment of three core assumptions [15]: (1) The first assumption states that the selected genetic instrument has to be truly associated with the exposure of interest, i.e., 25(OH)vitamin D. By selecting our instrument based on large-scaled GWAS and focusing only on genome-wide significant SNPs for 25(OH)vitamin D, we ensured that this first assumption was fulfilled. (2) If there is an effect of the genetic instrument on the outcome, besides the effect of 25(OH)vitamin D, it ss no longer possible to distinguish between the effects in an MR study. Accordingly, the second assumption states that the genetic instrument has to be independent of the outcome, conditional on the exposure and confounders of the exposure–outcome association [26]. (3) The third assumption states that the genetic instrument must not be associated with any confounder of the exposure–outcome relationship. Assumptions 2 and 3 are difficult to test, because it also includes associations with unknown confounders. Therefore, we investigated whether there was horizontal pleiotropy, i.e., genetics had an effect on DS or BD, besides its effect on 25(OH)vitamin D, by estimating the coefficients of Egger’s regression for MR, and checking whether the intercept was significantly unequal to zero.

### 2.3. Statistical Analysis

All tests were performed using the statistical software “R” version 3.5.2 (R Foundation for Statistical Computing, Vienna, Austria). Since the two outcomes were analyzed separately, the overall significance level of 0.05 was applied for each outcome, in order to control for multiple testing in the sense of a family-wise error rate.

To ensure that our analyses were not based on weak instruments, the F-statistics were computed. Following general recommendations, F-statistics above 10 indicate that the instruments were sufficiently strong. Given no evidence for horizontal pleiotropy, the summary data were analyzed for the single SNPs, as well as the combination of all SNPs as IV, using two MR methods (inverse variance weighted regression (IVW) and Egger’s regression). Other MR methods (simple mode, weighted mode and weighted median) were applied, with similar results (not shown). To visualize the results, forest and scatter plots were used, which combined the results of single and multi SNP analyses. In the forest plots, the single SNP effect estimates were displayed beside the multi SNP effect estimates, with the corresponding 95% confidence intervals. In the scatter plots, the single SNP effects on the exposure were plotted against the single SNP effects on the outcome (with corresponding standard deviation in both directions) and the estimated regression lines of the multi SNP analyses were added.

To examine whether the analyses were driven by any single SNP, sensitivity analyses using a leave-one-out approach were conducted. For this purpose, all SNPs but one were analyzed, using the IVW regression. In general, if the analyses are not driven by one single SNP, the regression coefficients remain relatively stable. Finally, we estimated the power to detect a true causal effect of a relative difference between 1% and 20%, per 1 standard deviation in 25(OH)vitamin D, which corresponded to the odds ratio values between 0.8 and 1.2 in 0.01 steps, with the approach proposed by Brion et al. [27]. Results from our analysis of the German representative KiGGS study might give a crude impression on the standard deviation of 25(OH)vitamin D in a healthy sample. In this analysis, we observed a standard deviation of 25.0 nmol/l and 25.5 nmol/l, in boys and girls aged 3–17 years [5].

## 3. Results

**Figure 1:**
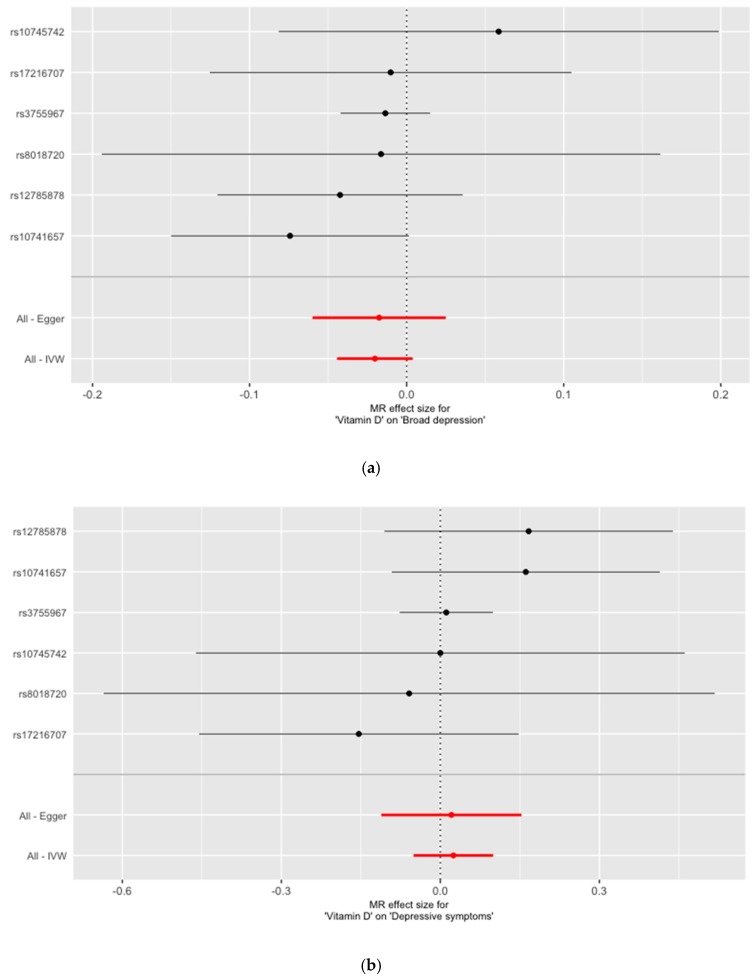
Results of the single and multi SNP analyses for the SNP effect of natural-log transformed 25(OH)vitamin D on (**a**) broad depression, and (**b**) depressive symptoms. The black lines visualize the results of single SNP analyses, the red lines visualize the results of the multi SNP analysis.

**Figure 2:**
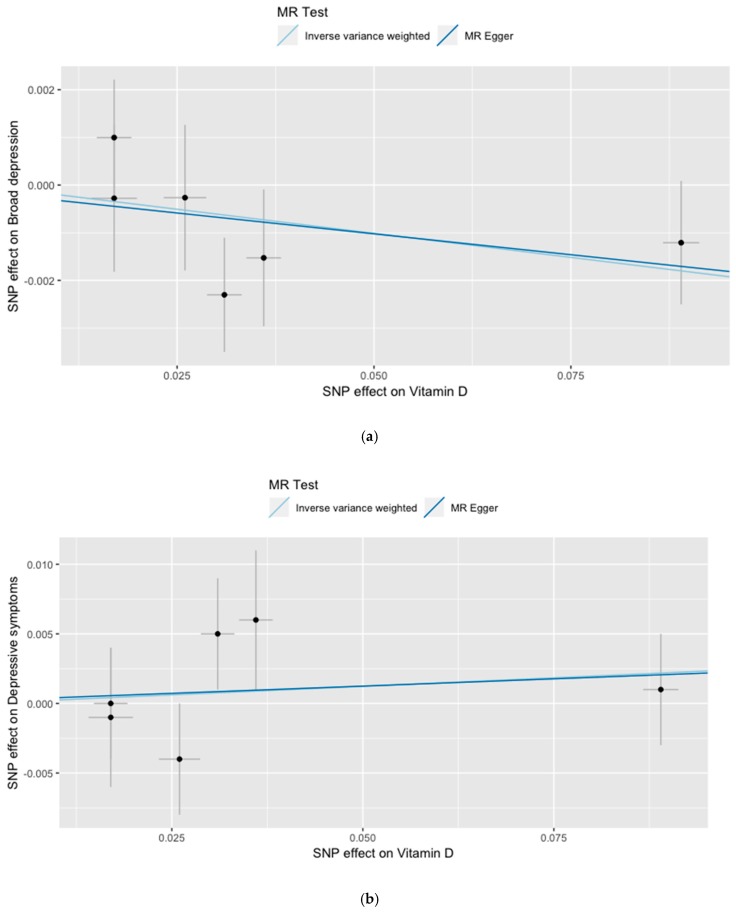
Results of the single and multi SNP analyses on the association between natural-log transformed 25(OH)vitamin D concentration and (**a**) broad depression and (**b**) depressive symptoms. A SNP effect of 0.025 means that carrying an alternative allele at this SNP was associated with an expected increase in the natural-log transformed 25(OH)D by 0.025; e^(beta) gives the change in 25(OH)vitamin D in percentage. Accordingly, a SNP effect of 0.025 was associated with an expected increase of 25(OH)D by 2.5% (e^0.025 = 1.025), while a SNP effect of 0.1 was associated with an expected increase of 25(OH)D by 10.5% (e^0.1 = 1.105).

The information on the association of the six selected SNPs with 25(OH)vitamin D concentrations, DS, and BD are presented in Table 1. None of the six 25(OH)vitamin D-lowering alleles were either associated with DS or with BD. With F = 381.0103 the F-statistic indicated strong instrumental variables. The overall estimates, calculated by IVW or MR Egger, did not reveal associations between 25(OH)vitamin D concentrations and DS or BD (Table 2, Figure 1 and Figure 2). Sensitivity analyses using the leave-one-out approach confirmed the lack of associations (Supplemental Tables S1 and S2). There was no evidence for pleiotropy either for DS (MR-Egger intercept: 0.0002; *p* = 0.949) or BD (MR-Egger intercept: −0.0001; *p* = 0.886). Power analyses revealed that our MR analyses had a 100% power to detect an OR of 1.1 for BD per 1 standard deviation decrease in natural-log transformed 25(OH)vitamin D (Supplemental Figure S1).

## 4. Discussion

Using data from the large-scaled GWAS in subjects from European descent, this MR study did not provide evidence for the suggested role of (genetically determined) 25(OH)vitamin D concentrations in the onset of DS or BD. Combined with the results from a previous MR study, based on a subsample of the data sets used for this current study, which showed no association with MDD, it might be hypothesized that raising of vitamin D concentrations seems to have no discernible preventive effect on the onset of depressive disorders in individuals of European descent.

In contrast to this conclusion, a meta-analysis of observational studies that compared the lowest and the highest category of vitamin D concentrations in adults, revealed an OR of 1.31 (95% confidence interval (CI) 1.0–1.71, *p* = 0.05) for depression derived from nine cross-sectional studies and a hazard ratio of 2.21 (95% CI 1.40–3.49, *p* = 0.0007) from three cohort studies [28]. We recently confirmed these findings in children using cross-sectional data of the representative KiGGS study in Germany, which illustrated an inverse association between 25(OH)vitamin D concentrations and emotional problems, measured by the Strengths and Difficulties Questionnaire (SDQ) [5]. A prospective analysis of the Avon longitudinal study for parents and children (ALSPAC) also seemed to indicate a preventive role of vitamin D in adolescence. While 25(OH)vitamin D concentrations at the age of 10 years were not associated with self-reported DS at the age of eleven years, long-term analyses revealed that those participants with 25(OH)D <50 nmol/l at ten years had a 20% increased risk of self-reported depressive symptoms at 14 years [7]. Overall, these associations from observational studies stand in contrast to the MR study on MDD [16], and stand also in contrast to our findings on DS and BD. As suggested by König and Del Greco, reverse causation and residual confounding should be considered as alternative explanations, if MR studies do not support findings from observational studies [15]. In fact, low vitamin D levels could well be a consequence of an already developing depression, e.g., in case of social withdrawal and reduced sunlight exposure (reverse causation). Residual confounding also seems reasonable considering that decreasing vitamin D concentrations were discussed to be a biomarker for impaired general health [29]. Accordingly, low vitamin D concentrations could be associated with a number of potential confounds, which hamper a complete adjustment in the statistical analyses of observational studies.

A limitation of our MR study was that the analyzed SNPs only explained 2.8% of the variance of vitamin D concentrations. Accordingly, the lacking association between the respective SNPs and the development of depression might be confounded by the weak effect of these genetic determinants on the vitamin D level (weak instrument bias). However, the F-Statistics indicated that the genetic instruments used were sufficiently strong.

The considered GWAS meta-analyses for vitamin D, DS, and BD for MR analysis were all restricted to individuals of European descent. Since our MR analysis relied on the provided information in the underlying GWAS meta-analyses, it was, for example, not possible to conduct stratified analyses for different countries, ethnicities, or age groups. Accordingly, the effects of vitamin D concentrations observed in this MR study might not be transferable to populations with other specific characteristics (e.g., ethnicity and age). Furthermore, horizontal pleiotropy, i.e., an association between the MR instrument and the outcome of interest via pathways other than the suggested exposure, is a general issue in MR studies and is a source of bias [14]. Although pleiotropy cannot be completely ruled out in our MR study, the MR Egger intercept revealed no indication of pleiotropy. Another limitation is that the statistical power only allowed the detection of OR > 1.1 per 1 standard deviation decrease in the natural-log transformed 25(OH)vitamin D with a statistical power of 100% (OR > 1.07 with 80% power). Accordingly, small preventive effects below this threshold could not completely be ruled out. Additionally, it must be kept in mind that DS and BD represent a broad definition of depression. As depression is a heterogenic disorder, vitamin D might still be effective in specific subtypes of depression. Finally, it has to be considered that vitamin D represents only one of numerous etiological factors in the complex etiological model of depression, including endocrinology, inflammation, and neurotransmission pathways [17]. At least, at the population level, the role of vitamin D in this process might be of minor importance.

## 5. Conclusions

This MR study did not support the suggested preventive role of 25(OH)vitamin D concentrations in the development of depression. Other health and lifestyle factors related to low 25(OH)vitamin D concentrations, such as insufficient sunlight exposure as a consequence of an already beginning depression, could explain the observed associations from observational studies.

## Figures and Tables

**Table 1:** Genome-wide significant single nucleotide polymorphisms (SNPs) for natural log-transformed 25(OH)vitamin D concentrations and their association with broad depression and depressive symptoms.

					Association with Natural Log-Transformed 25(OH)vitamin D			Association with Broad Depression			Association with Depressive Symptoms		
SNP	Chromosome	Gene	Effect/Reference Allele	AF *	Effect Estimate (Beta) #	SE	*p*	Effect Estimate (Beta)	SE	*p*	Effect Estimate (Beta)	SE	*p*
rs3755967	4	GC	T/C	0.28	−0.089	0.0023	4.74E–343	0.0012	0.0013	0.350	−0.001	0.004	0.731
rs10741657	11	CYP2R1	A/G	0.4	0.031	0.0022	2.05E–46	0.002	0.001	0.055	0.005	0.004	0.309
rs12785878	11	NADSYN1_DHCR7	T/G	0.75	0.036	0.0022	3.80E–62	−0.002	0.001	0.287	0.006	0.005	0.215
rs10745742	12	AMDHD1	T/C	0.4	0.017	0.0022	1.88E–14	0.001	0.001	0.412	0.000	0.004	0.976
rs8018720	14	SEC23A	C/G	0.82	−0.017	0.0029	4.72E–09	0.0003	0.0015	0.857	0.001	0.005	0.780
rs17216707	20	CYP24A1	T/C	0.79	0.026	0.0027	8.14E–23	0.0003	0.0015	0.862	−0.004	0.004	0.340

AF: allele frequency; SE: standard error; * information on allele frequency were derived from [18]. # Calculating e^(beta) gives the change in 25(OH)vitamin D in percentage. For instance, for SNP rs3755967, e^(−0.089) renders 0.91, meaning that carriage of the alternative allele reduces 25(OH)D by 9%.

**Table 2:** Results of the Mendelian Randomization analyses on the associations of natural-log transformed 25(OH)vitamin D concentrations with broad depression and depressive symptoms.

	Association of 25(OH)vitamin D with Broad Depression			Association of 25(OH)vitamin D with Depressive Symptoms		
SNP	Effect Estimate (Beta)	SE	*p*	Effect Estimate (Beta)	SE	*p*
rs3755967	−0.0136	0.0145	0.350	0.0112	0.0450	0.803
rs10741657	−0.0743	0.0386	0.055	0.1613	0.1290	0.211
rs12785878	−0.0424	0.0398	0.287	0.1667	0.1389	0.230
rs10745742	0.0586	0.0715	0.412	0.0000	0.2353	1.000
rs8018720	−0.0163	0.0907	0.857	−0.0588	0.2941	0.841
rs17216707	−0.0102	0.0587	0.862	−0.1538	0.1538	0.317
All—Inverse variance weighted (IVW)	−0.0202	0.0123	0.099	0.0246	0.0384	0.521
All—MR Egger	−0.0175	0.0217	0.464	0.0209	0.0674	0.772

SE: standard error; SNP: single nucleotide polymorphisms.

## Supplementary Materials

The following are available online at https://www.mdpi.com/2072-6643/11/5/1085/s1, Table S1: Sensitivity analyses using the leave-one-out approach on the association of natural-log transformed 25(OH)vitamin D concentrations with broad depression, Table S2: Sensitivity analyses using the leave-one-out approach on the association of natural-log transformed 25(OH)vitamin D concentrations with depressive symptoms. Figure S1. Calculated power to detect a true causal effect of a relative difference between 1% and 20% per 1 standard deviation in 25(OH)vitamin D.
