# Supplementary material for: Vitamin D and the Risk of Depression: A Causal Relationship? Findings from a Mendelian Randomization Study
Source: Nutrients. 2019 May 16;11(5):1085. doi: 10.3390/nu11051085 (PMC6566390; doi:10.3390/nu11051085)
Supplement: Supplementary file 1 [file nutrients-11-01085-s001.pdf]

Table S1. : Sensitivity analyses using the leave-one-out approach on the association of natural-log transformed 25(OH)vitamin D concentrations with broad depression

SNP	b	se	p
Inverse variance weighted results including all SNPs	-0.0202270	0.0122753	0.0993989
rs10741657	-0.0141555	0.0129465	0.2742275
rs10745742	-0.0226221	0.0124604	0.0694448
rs12785878	-0.0178997	0.0129029	0.1653628
rs17216707	-0.0206856	0.0125526	0.0993709
rs3755967	-0.0366862	0.0228975	0.1091140
rs8018720	-0.0202996	0.0123893	0.1013221

Table S2. : Sensitivity analyses using the leave-one-out approach on the association of natural-log transformed 25(OH)vitamin D concentrations with depressive symptoms

SNP	b	se	p
Inverse variance weighted results including all SNPs	0.0246156	0.0383811	0.5212968
rs10741657	0.0113490	0.0402007	0.7777063
rs10745742	0.0252885	0.0389021	0.5156571
rs12785878	0.0128709	0.0399362	0.7472365
rs17216707	0.0364600	0.0396343	0.3576190
rs3755967	0.0606584	0.0737661	0.4109019
rs8018720	0.0260611	0.0387121	0.5008179

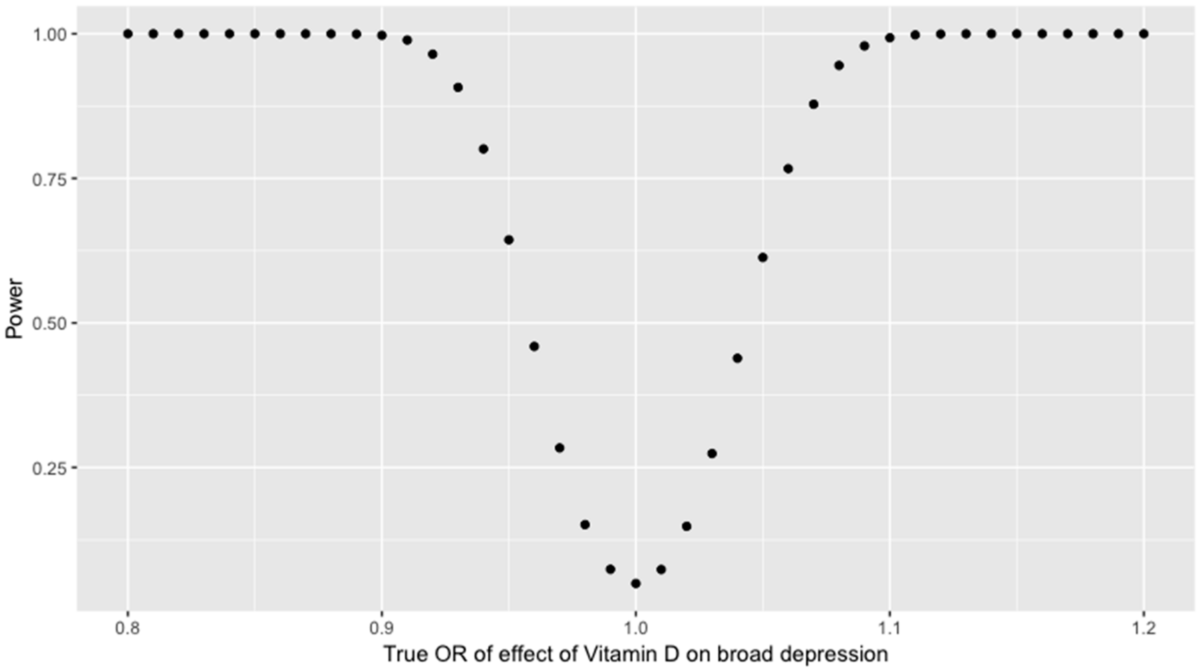

Figure S1. Calculated power to detect a true causal effect of a relative difference between 1% and 20% per 1 standard deviation in 25(OH)vitaminD.
